# Mechanism of Lethal Toxin Neutralization by a Human Monoclonal Antibody Specific for the PA_20_ Region of *Bacillus anthracis* Protective Antigen

**DOI:** 10.3390/toxins3080979

**Published:** 2011-08-09

**Authors:** Donald Reason, Justine Liberato, Jinying Sun, Jessica Camacho, Jianhui Zhou

**Affiliations:** Children’s Hospital Oakland Research Institute, Oakland, CA 94609, USA; Email: justine77@gmail.com (J.L.); jsun@chori.org (J.S.); Jessica.Camacho@fda.hhs.gov (J.C.); zhou.jianhui@gene.com (J.Z.)

**Keywords:** anthrax, *Bacillus anthracis*, antibody epitope, paratope, human monoclonal antibody, protective antigen, furin, toxin neutralization

## Abstract

The primary immunogenic component of the currently approved anthrax vaccine is the protective antigen (PA) unit of the binary toxin system. PA-specific antibodies neutralize anthrax toxins and protect against infection. Recent research has determined that in humans, only antibodies specific for particular determinants are capable of effecting toxin neutralization, and that the neutralizing epitopes recognized by these antibodies are distributed throughout the PA monomer. The mechanisms by which the majority of these epitopes effect neutralization remain unknown. In this report we investigate the process by which a human monoclonal antibody specific for the amino-terminal domain of PA neutralizes lethal toxin in an *in vitro* assay of cytotoxicity, and find that it neutralizes LT by blocking the requisite cleavage of the amino-terminal 20 kD portion of the molecule (PA_20_) from the remainder of the PA monomer. We also demonstrate that the epitope recognized by this human monoclonal does not encompass the _166_RKKR_169_ furin recognition sequence in domain 1 of PA.

## 1. Introduction

The *Bacillus anthracis* binary toxins are major virulence factors in anthrax infection [1,2]. The cell surface recognition element of this toxin system is an 83-kDa secreted protein known as protective antigen (PA). PA is the primary immunogenic component in the anthrax vaccine currently licensed for use in the United States (BioThrax™ or AVA, Emergent Biosystems), and ongoing attempts to develop a “next generation” anthrax vaccine are relying on a recombinant form of PA as the sole immunogenic constituent. Efforts towards the design of more efficacious anthrax vaccines would benefit from a more thorough understanding of both the biology of this protein toxin, and the immunobiology of its interaction with the immune system of the vaccinated or infected host.

We have used repertoire profiling to de-convolute the polyclonal human antibody response to PA into its component PA-specific paratopes [3,4,5,6]. We find that PA activates a diverse collection of B cells which utilize a variety of variable heavy, variable light, diversity, and joining gene segments to form PA-specific antibody [6]. Descendants of these clones undergo expansion, somatic hypermutation, and class switch recombination. Together, the ability of PA to both recruit a diverse B-cell population and drive significant somatic maturation gives rise to a complex serum antibody response composed of multiple sequence-unique clonotypes. The amino-terminal region of PA_83_ (PA_20_) is immunodominant in humans. Although PA_20_ comprises less than 25% of the mass of the monomer, over 60% of the PA-specific antibody response recognizes epitopes associated with this region. This epitope dominance has been demonstrated in both the polyclonal human serum response following vaccination, and in the monoclonal antibodies isolated from vaccinated donors [4]. Individual antibodies capable of neutralizing lethal toxin (LT)* in vitro* are relatively infrequent in vaccinated individuals, and constitute only about 24% of the PA-specific paratopes isolated. And, although neutralizing paratopes occur less frequently and are less effective among those individual antibodies recognizing PA_20_-associated epitopes, the immuno-dominance of this region results in a significant portion of the post vaccination LT-neutralizing potential of the antibody response being derived from PA_20_-specific paratopes [3]. 

PA_83_ is cleaved very rapidly in the host into free PA_20_ and cell-associated PA_63_ (which further associates to form PA_441_). Given that PA_20_ plays no direct role in LT-mediated toxicity, the presence of neutralizing epitopes in this region of the molecule is somewhat unexpected. In this report we determine the mechanism by which a human monoclonal antibody specific for PA_20_ neutralizes lethal toxin in an *in vitro* assay of cytotoxicity. As has been demonstrated for a murine monoclonal with similar binding and neutralization characteristics [7], this human antibody neutralizes LT by blocking the requisite cleavage of PA_20_ from the remainder of the PA monomer. Unlike murine monoclonal antibodies, the epitope recognized by this human monoclonal is distant from the furin recognition sequence in domain 1 of PA.

## 2. Materials and Methods

### 2.1. Human Monoclonal Antibody

Isolation of the PA-specific monoclonal antibody 47F12 from an AVA-vaccinated donor by repertoire cloning has been previously described [4]. This antibody was isolated as a recombinant FAB fragment in *E. coli* and subsequently expressed as an IgG1 antibody in CHO cells. Secreted antibody was concentrated from supernatant, quantitated by capture ELISA, and used in all subsequent assays. Other human monoclonal antibodies isolated from the same study were used as controls. These include 1A5 (PA_63_-specific, neutralizing), 11A11 (PA_20_-specific, non-neutralizing), 9G5 (PA_20_ specific, non-neutralizing), 4A12 (D4-specific, neutralizing), and 24B1 (PA_63_-specific, neutralizing).

### 2.2. Construction of PA_20_- and D4-GFP Fusion Proteins

The PA_20_ amino-terminal (residues 1–191) and the domain 4 (D4) carboxy-terminal (residues 587–735) portion of the PA monomer were cloned using PCR and expressed fused to intact green fluorescent protein (GFP). Cloning primers for the amino-terminal fragment were ATATGAATTCTATGGAAGTTAAACAGGAGAACCG (5') and ATATGGATCCTCCTTCTA-CCTCTAATGAATC (3'). Cloning primers for the D4 region were GCATTAGAATTCGCATCA CCATCACCATCACATGAATATTTTAATAAGAGATAAACG (5') and CGTATATCTAGAAGG-ATCCCCTATC TCATAGCCTTTTTTAGAAAAGAT (3'). Fusion proteins were expressed in *E. coli* and purified by nickel-chelate chromatography. 

### 2.3. Domain Specificity of PA-Specific Antibodies

The domain specificity of 47F12 was determined using dot blots. PA and PA-derived proteins were spotted onto nitrocellulose membranes using a 96-well manifold. The resulting membrane was then cut into strips containing one spot for each protein. Antibodies were incubated with the blots for 2 h at 37 °C, washed, and binding visualized by means of an alkaline-phosphatase conjugated goat antibody specific for human lambda light chains followed by BCIP/NBT color development.

### 2.4. *In vitro* Toxin Neutralization Assay

PA and LF were purchased from List Biological Laboratories, Carlsbad, CA. RAW 264.7 cells were plated at 4 × 10^4^ cells per well (in 65 μL assay medium) in Nunclon Delta 96-well plates and incubated at 37 °C, 5% CO_2_ for approximately 4 to 5 h to ensure proper settling and attachment. Antibodies and PA/LF were pre-incubated in Greiner Bio-One 96-well plates at 37 °C, 5% CO_2_ for 1 h, and then were transferred to the 96-well plate containing RAW 264.7 cells. The plate was then placed back into the incubator at 37 °C, 5% CO_2_ for overnight incubation. The following morning, 20 μL Cell-Titer Blue reagent (Promega) was added to each well of the assay plate. Optical density (at 570/595 nm) was determined for each well four hours later using a microtiter plate reader.

### 2.5. Domain-Specific Blockade of Neutralizing Antibodies in the RAW Cell Assay

Neutralizing antibodies were pre-incubated with modified forms of PA to verify the specificity of the antibody-PA binding in the toxin neutralization assay. PA_20_ (residues 1–191) was expressed fused to GFP as described above and in [3]. A non-functional mutant of PA (PArb-) was constructed using QuickChange™ Mutagenesis (Stratagene) by mutating two residues in the D4 region of wild type PA (N682A and D683A) to remove its ability to bind to the cell surface receptor. This modification was necessary to prevent PA added to the assay as an inhibitor from participating in LF-mediated toxicity. Neutralizing antibodies were incubated with the modified PA constructs overnight at concentrations sufficient for 50% inhibition of LT-mediated cytotoxicity. These were then added to the neutralization assay described above, and the degree to which the pre-incubation blocked the antibodies ability to neutralize toxin was calculated.

### 2.6. Furin Digestion

To determine the ability of PA_20_-specific monoclonal antibodies to inhibit the proteolytic cleavage of PA by furin, ^125^I-PA was incubated with individual monoclonal antibodies for 1 h at 37 °C followed by purification of the antibody-PA complexes with Protein A magnetic beads. Complexed ^125^I-PA was incubated with furin (New England Biolabs) in the appropriate buffer (100 mM tris, pH 7.5, 0.5% Triton X-100, 1 mM CaCl_2_, 1 mM 2-mercaptoethanol) for 10 min at 37 °C. Loading buffer was then added to each sample, the samples subjected to SDS-PAGE, and the size of the separated ^125^I-PA determined using a phosphoimager.

### 2.7. ^125^I-PA Recovery Assay

RAW 264.7 cells were plated at 5 × 10^5^ cells per well in 24-well plates and incubated at 37 °C, 5% CO_2_ overnight. Antibodies to be tested, ^125^I-PA, and LF were pre-incubated in tubes at 37 °C for 1 h and then were transferred into the 24-well plate containing RAW 264.7 cells. The plate was then placed back into the incubator at 37 °C, 5% CO_2_ for 1 h (cell lysis begins at approximately 3 h). Media was then aspirated from each well, adherent cells washed 5 times with medium, and lysed in 0.150 mL lysis buffer (1%NP-40, 0.25% Sodium deoxycholate, 150 mM NaCl, 1 mM EDTA, 2 mM MgCl_2_ in Tris-HCL pH 7.4). Lysis buffer was then collected from each well and subjected to SDS-PAGE analysis as described above for the furin digest assay.

### 2.8. Epitope Mapping Using Yeast Display

PA_20_, truncated derivatives of PA_20_, and randomly mutated PA_20_ were displayed on the surface of yeast as Aga2 fusion proteins as described by Boder and Wittrup [8]. The vector pYD1 and the host yeast strain EBY100 were purchased from Invitrogen (Carlsbad, CA, USA). PA_20_ (residues 1–191) and truncated PA_20_ (residues 1–140) were amplified by PCR and inserted into the BamH I/ Xho I restriction sites in pYD1 (in frame with AGA2 and separated from it by a flexible linker). Randomly mutated libraries of PA_20_ were generated by error-prone PCR. MnCl_2_ concentration were titrated, and conditions selected such that the average number of amino acid substitutions per molecule ranged from 1 to 3. These fragments were ligated into pUC18, expanded in *E. coli*, excised, and inserted into the BamH I/ Xho I restriction sites of pYD1. Following sequence verification, yeast were transformed with the plasmid, expanded overnight at 30 °C, transferred into Galactose-containing YNB medium (Yeast Nitrogen Base w/o amino acids (Difco)) to induce recombinant protein expression, and incubated for 48 h at 20 °C. For PA_20_ and the truncated derivatives of PA_20_, yeast were allowed to bind to the 47F12 antibody, and binding detected by a secondary phycoerythrin-conjugated anti-light chain antibody followed by flow cytometry. For the randomly mutated libraries, bulk yeast cultures were stained with 47F12 and a second non-competing PA_20_ specific antibody. Cell sorting was performed to select those yeast clones that had lost the ability to bind 47F12 but retained binding to the second antibody (this verifies surface expression of the construct). These clones were re-grown, their binding profiles verified, and the sequence of the PA_20_ insert they contained determined to identify those substitutions that had resulted in the loss of the 47F12 epitope. Approximately 40 binding loss mutants were analyzed to identify the 47F12 epitope. Residues comprising the epitope were mapped onto the surface of the solved structure of PA [9]. The region containing the furin recognition sequence (residues 162–174) is not resolved in the solved structure, and was modeled using the SWISS-MODEL homology server for inclusion in Figure 7 [10].

### 2.9. Antibody Concentration and Binding Assays

Antibody concentration was determined by a capture ELISA in which goat anti-human Fc (The Binding Site, Birmingham, UK) immobilized on a microtiter plate captures IgG, which is then detected by alkaline-phosphatase labeled goat anti-human L chain (Biosource International, Camarillo, CA, USA). This assay is standardized using a purchased IgG1 protein standard (Sigma). PA binding in ELISA was determined for IgG1 antibodies on 96-well plates coated with 5 μg/mL PA_83_ and developed with alkaline-phosphatase conjugated goat antibody specific for human lambda light chains. The Diphtheria toxin mutant CRM197 was used as a specificity control for the binding assay.

## 3. Results

**Figure 1:**
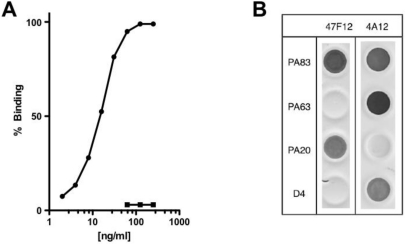
PA-specific binding and domain specificity of the human monoclonal antibody 47F12. (**A**) Concentration dependent binding of 47F12 to immobilized PA (circles) and the irrelevant toxin CRM197 (squares) in an ELISA assay; (**B**) Domain specificity of binding by the human monoclonal antibodies 47F12 and 4A12. PA_83_ and PA_63_ were purchased from List Biological Laboratories. PA_20_ and domain 4 (D4) were expressed as a fusion proteins with GFP and purified in our laboratory.

**Figure 2:**
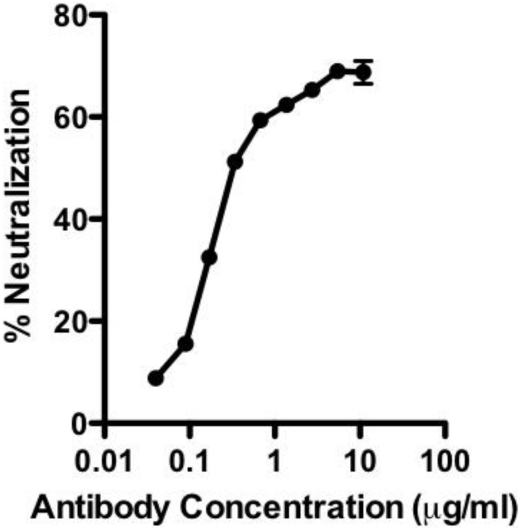
Neutralization of lethal toxin in the RAW 264.7 cytotoxicity assay by the human monoclonal antibody 47F12.

**Figure 3:**
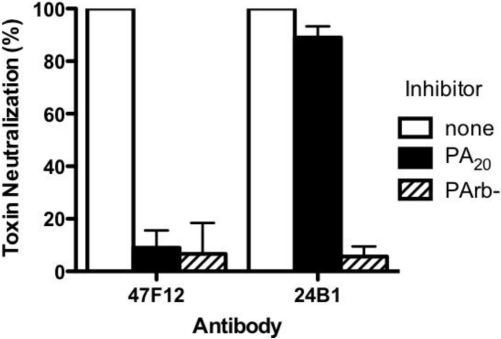
Inhibition of Lethal Toxin neutralization. PA_20_-specific (47F12) and PA_63_-specific (24B1) neutralizing monoclonal antibodies were incubated with PA_20_ or PArb- prior to their addition to the RAW cytotoxicity assay. The degree to which pre-incubation with the toxin components blocked the antibodies’ ability to neutralize lethal toxin was calculated as a fraction of the neutralization achieved when antibody was pre-incubated with buffer alone.

The human monoclonal antibody 47F12 was isolated from a donor vaccinated with the currently licensed anthrax vaccine (BioThrax) as part of a larger study of the human PA-specific antibody repertoire [4]. 47F12 binds PA in a concentration dependent manner (Figure 1A) and is specific for an epitope in the PA_20_ amino-terminal region of the molecule (Figure 1B). When tested in the lethal toxin neutralization assay, the antibody neutralized lethal toxin in a dose dependent manner, with 50% neutralization (NC_50_) occurring at 230 ng/mL (Figure 2). The maximum degree of neutralization achievable in the assay was about 74%. The inability to achieve 100% toxin neutralization in the RAW 264.7 assay is a characteristic of PA_20_-specific neutralizing antibodies in general, and may result from competition in the assay by free PA_20_ that is cleaved from PA_83_ in the course of intoxication. The ability of 47F12 to neutralize lethal toxin was specifically inhibited both by PA_83_ and PA_20_, demonstrating that the PA_20_-associated epitopes recognized in the direct binding assays were also those responsible for toxin neutralization (Figure 3). Together these data indicate that the PA-specific human monoclonal antibody 47F12 functions to neutralize lethal toxin by binding to an epitope present in the PA_20_ region of the PA monomer.

**Figure 4:**
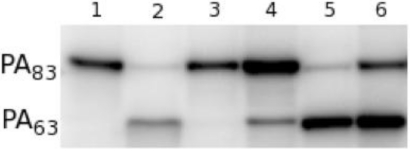
Furin proteolysis of ^125^I-PA *in vitro*. (1) 30 ng ^125^I-PA; (2) PA + furin; (3) PA + furin + EDTA; (4) PA pre-incubated with PA_20_-specific neutralizing antibody 47F12; (5) PA pre-incubated with PA_63_-specific neutralizing antibody 1A5; (6) PA pre-incubated with PA_20_-specific non-neutralizing antibody 9G5.

Since PA_20_ lacks all of the residues involved in cell surface binding, we postulated that the antibody achieved toxin neutralization by inhibiting the requisite cleavage of PA_20_ from the remainder of the molecule by furin. In the presence of furin, radio-labeled PA_83_ is rapidly cleaved at the _166_RKKR_169_ recognition sequence to yield PA_63_ (Figure 4, lane 1, 2) [11,12]. When radio-labeled PA_83_ is pre-incubated with 47F12, cleavage is significantly reduced (Figure 4, lane 4). Pre-incubation with the PA_63_-specific neutralizing antibody 1A5 does not result in the blockade of furin-mediated cleavage (Figure 4, lane 5), nor does incubation with the PA_20_-specific non-neutralizing antibody 9G5 (Figure 4, lane 6). These data indicate that 47F12 does in fact interfere with the furin mediated cleavage of PA_83_, and that this interference is epitope specific, since 9G5, which is also specific for PA_20_, fails to inhibit the enzyme.

**Figure 5:**
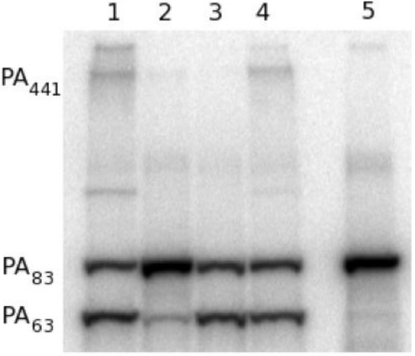
Analysis of ^125^I-PA recovered from *in vitro* RAW cell culture. (1) Lethal toxin alone added to cell culture; (2) Lethal toxin pre-incubated with the PA_20_-specific neutralizing antibody 47F12 prior to addition to the cell culture; (3) Lethal toxin pre-incubated with the PA_63_-specific neutralizing antibody 1A5; (4) Lethal toxin pre-incubated with the PA_20_-specific non-neutralizing antibody 11A11; (5) 1 ng ^125^I-PA.

To determine if 47F12 was functioning in a similar manner in the RAW 264.7 neutralization assay, lethal toxin incorporating radio-labeled PA was added to the cultures, recovered following incubation, and its size determined using SDS-PAGE (Figure 5). When no antibody was added to the assay, PA_63_ (indicating cleavage), PA_441_ (indicating heptamer formation), and residual PA_83_ were recovered following 1 h of incubation (Figure 5, lane 1). Pre-incubation of lethal toxin with 47F12 prior to its addition to the assay results in the recovery of PA_83_ alone, indicating that both cleavage and subsequent heptamer formation had been inhibited (Figure 5, lane 2). Pre-incubation with the PA_63_-specific neutralizing monoclonal antibody 1A5 did not block cleavage, but did inhibit the formation of the 441-kDa heptamer (Figure 5, lane 3). Pre-incubation of lethal toxin with the non-neutralizing, PA_20_-specific antibody 11A11 blocked neither cleavage nor heptamer formation (Figure 5, lane 4). These data indicate that 47F12 blocks the furin-mediated cleavage of PA_83_ required for lethal toxin-mediated cytotoxicity in the RAW 264.7 neutralization assay, and that this inhibition is epitope specific as well, since the PA_20_-specific non-neutralizing antibody 11A11 did not interfere with proteolysis.

**Figure 6:**
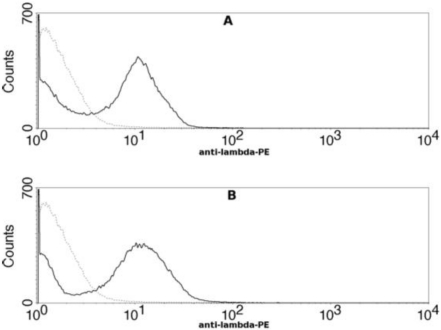
Flow cytometric analysis of human monoclonal antibody 47F12 binding to yeast cells displaying PA_20_ or truncated PA_20_ on their surface. (**A**) 47F12 binding to yeast cells displaying PA_20_ (residues 1–191) on their surface; (**B**) 47F12 binding to yeast cells displaying truncated PA_20_ (residues 1–140) on their surface. Binding was detected with a phycoerythrin-conjugated antibody specific for human lambda light chains.

To determine if the epitope recognized by 47F12 encompassed the _166_RKKR_169_ furin recognition sequence, a truncated deletion mutant of PA_20_ was constructed, displayed on the surface of yeast, and tested for antibody binding using flow cytometry. The ability of 47F12 to bind to a fragment of PA_20_ that was truncated at residue 140 did not differ from binding to the full length PA_20_ domain (Figure 6). Therefore, although 47F12 blocks cleavage by furin at the _166_RKKR_169_ recognition sequence, the epitope recognized by this antibody does not contain the RKKR recognition sequence itself, nor is it found within the 27 residue region upstream from the recognition motif. 

**Figure 7:**
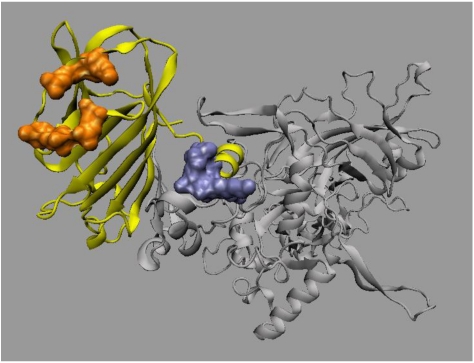
Residues recognized by the the human monoclonal antibody 47F12 mapped onto the solved structure of PA_83_[9]. The PA_20_ region of the monomer is in yellow, the remainder of the monomer is in gray, residues comprising the epitope (E_95_, N_98_, A_100_, N_104_, I_106_) highlighted in orange, and the furin recognition site shown in blue.

To identify the PA_20_ residues required for 47F12 binding, a yeast library displaying randomly mutated PA_20_ on its surface was screened using flow cytometry and those yeast clones that had lost the ability to bind 47F12 sorted and their inserts sequenced. Binding loss mutants were selected for analysis only if they retained the ability to bind the non-competing antibody 18C12. This verifies that loss of 47F12 binding is not due to a mutation that blocks surface expression of the PA_20_ molecule. Analysis of 40 binding loss mutants identified substitutions at residues E_95_, N_98_, A_100_, N_104_, and I_106_ that resulted in the loss of affinity of PA_20_ for 47F12. When mapped to the solved structure of PA, these residues were co-located on the surface of the molecule and distant from the _166_RKKR_169_ recognition sequence (Figure 7).

## 4. Discussion and Conclusions

The mechanism by which antibodies neutralize lethal toxin is not completely understood. Neutralizing and non-neutralizing epitopes are distributed throughout the PA monomer, and the majority of neutralizing epitopes recognized by vaccinated humans reside in regions other than those involved with receptor interactions, implying that the general assumption [13,14] that neutralizing antibodies block PA’s ability to bind to its cognate receptor is incomplete. The additional discovery that the majority of individual PA-specific antibody clonotypes do not neutralize toxin in spite of avid antigen binding suggest that it is the specific epitope bound, rather than overall affinity for the antigen, that determines an antibodies’ ability to neutralize toxin function [3]. The mechanism by which the majority of toxin neutralization is realized therefore remains to be determined.

The series of events required for lethal toxin to result in cell death is complex [1,2,15,16,17]. PA must bind to the cell surface, undergo proteolytic cleavage, heptamerize to form the pre-pore, bind lethal factor (LF), insert into the membrane, and be endocytosed. It is likely that antibody-mediated neutralization involves the disruption of one or more of these requisite steps in the intoxication process. Neutralizing antibodies within the PA_20_-specific portion of the antibody repertoire are both infrequent and much less efficient than those recognizing PA_63_, but they are nevertheless well represented due the overall immunodominance of PA_20_-associated epitopes in the human response [4]. This report demonstrates that the monoclonal antibody 47F12 blocks the ability of furin to cleave, and thereby activate, PA. Although the PA_20_ peptide itself has no known role in toxicity, its proteolytic removal is an absolute requirement for PA heptamerization and LF binding [18]. Other PA_20_-specific neutralizing antibodies may function by interfering with other steps in the intoxication pathway. For example, following cleavage, PA_20_ remains non-covalently associated with PA_63_, and this association must be disrupted in order for those residues involved in the self-assembly of the heptamer to be exposed [9]. An antibody that blocked the disassociation of PA_20_ from PA_63_ may also inhibit intoxication without blocking furin cleavage directly. We have identified two additional neutralizing epitopes within PA_20_ recognized by human PA-specific monoclonal antibodies, neither of which include the _166_RKKR_169_ recognition sequence. The mechanism by which these antibodies neutralize PA has not yet been determined.

The relative contribution of antibodies specific for different regions of the PA monomer to toxin neutralization *in vivo* is unknown. In animal models, both PA_20_ and PA_63_ have been shown to circulate following infection [19,20], and it is reasonable to assume that antibodies specific for these two regions would complex with their respective epitopes in the circulation. The implications for this complex formation on toxin neutralization differ, however, according to the region of the molecule bound. Antibodies specific for PA_20_-associated epitopes can only effect neutralization while PA_20_ remains associated with PA_63_. Circulating PA_20_ would therefore compete with cell-bound PA_20_ for neutralizing paratopes, thereby lowering the overall efficacy of antibodies directed against neutralizing epitopes in the amino-terminal region of the PA monomer. Neutralizing antibodies binding circulating PA_63_ would, on the other hand, impede toxicity whether the determinants are encountered in solution or at the cell surface. The *in vivo* performance of the murine PA_20_-specific monoclonal 7.5G, which efficiently neutralizes lethal toxin *in vitro*, but is only modestly effective *in vivo*, would support this hypothesis [7]. The paucity of neutralizing paratopes within the immuno-dominant PA_20_-specific portion of the response, the relative inefficiency of those that do occur, and their susceptibility to inhibition by circulating PA_20_ in the host, when considered together, may partially account for the relatively poor performance of AVA in vaccine recipients.

## References

[1] Collier R.J., Young J.A. (2003). Anthrax toxin. Annu. Rev. Cell Dev. Biol..

[2] Leppla S., Moss J. (1995). Anthrax toxins. Bacterial Toxins and Virulence Factors in Disease.

[3] Reason D., Liberato J., Sun J., Keitel W., Zhou J. (2009). Frequency and domain specificity of toxin-neutralizing paratopes in the human antibody response to the anthrax vaccine AVA. Infect. Immun..

[4] Reason D.C., Ullal A., Liberato J., Sun J., Keitel W., Zhou J. (2008). Domain specificity of the human antibody response to *Bacillus anthracis* protective antigen. Vaccine.

[5] Reason D.C., Zhou J. (2006). Codon insertion and deletion functions as a somatic diversification mechanism in human antibody repertoires. Biol. Direct.

[6] Zhou J., Ullal A., Liberato J., Sun J., Keitel W., Reason D.C. (2008). Paratope diversity in the human antibody response to *Bacillus anthracis* protective antigen. Mol. Immunol..

[7] Rivera J., Nakouzi A., Abboud N., Revskaya E., Goldman D., Collier R.J., Dadachova E., Casadevall A. (2006). A monoclonal antibody to *Bacillus anthracis* protective antigen defines a neutralizing epitope in domain 1. Infect. Immun..

[8] Boder E.T., Wittrup K.D. (1997). Yeast surface display for screening combinatorial polypeptide libraries. Nat. Biotechnol..

[9] Petosa C., Collier R.J., Klimpel K.R., Leppla S.H., Liddington R.C. (1997). Crystal structure of the anthrax toxin protective antigen. Nature.

[10] Arnold K., Bordoli L., Kopp J., Schwede T. (2006). The SWISS-MODEL Workspace: A web-based environment for protein structure homology modelling. Bioinformatics.

[11] Matthews D.J., Goodman L.J., Gorman C.M., Wells J.A. (1994). A survey of furin substrate specificity using substrate phage display. Protein Sci..

[12] Molloy S.S., Bresnahan P.A., Leppla S.H., Klimpel K.R., Thomas G. (1992). Human furin is a calcium-dependent serine endoprotease that recognizes the sequence Arg-X-X-Arg and efficiently cleaves anthrax toxin protective antigen. J. Biol. Chem..

[13] Drysdale M., Olson G., Koehler T.M., Lipscomb M.F., Lyons C.R. (2007). Murine innate immune response to virulent toxigenic and non-toxigenic *Bacillus anthracis* strains. Infect. Immun..

[14] Little S.F., Leppla S.H., Cora E. (1988). Production and characterization of monoclonal antibodies to the protective antigen component of *Bacillus anthracis* toxin. Infect. Immun..

[15] Brossier F., Mock M. (2001). Toxins of *Bacillus anthracis*. Toxicon.

[16] Brossier F., Weber-Levy M., Mock M., Sirard J.C. (2000). Role of toxin functional domains in anthrax pathogenesis. Infect. Immun..

[17] Mock M., Fouet A. (2001). Anthrax. Annu. Rev. Microbiol..

[18] Beauregard K.E., Collier R.J., Swanson J.A. (2000). Proteolytic activation of receptor-bound anthrax protective antigen on macrophages promotes its internalization. Cell. Microbiol..

[19] Ezzell J.W., Abshire T.G. (1992). Serum protease cleavage of *Bacillus anthracis* protective antigen. J. Gen. Microbiol..

[20] Moayeri M., Wiggins J.F., Leppla S.H. (2007). Anthrax protective antigen cleavage and clearance from the blood of mice and rats. Infect. Immun..

